# Elucidation of Response Mechanism of Potato to Nitrogen Stress by Physiological and Transcriptional Analyses

**DOI:** 10.3390/genes17030308

**Published:** 2026-03-05

**Authors:** Kaixin Ding, Ying Shan, Lichun Wang, Jiling Song, Mengping Yang, Yong Zhang, Lei Wang, Xuhong Sun, Mingxue Li, Guokui Tian, Fengyun Li, Haiyan Wang

**Affiliations:** 1 Keshan Branch of Heilongjiang Academy of Agricultural Sciences, Qiqihar 161000, China; ksfydkx@163.com (K.D.); ksfysy@163.com (Y.S.); ksfyymp@163.com (M.Y.); ksfyzy@163.com (Y.Z.); ksfywl@126.com (L.W.); ksfysuh@163.com (X.S.); ksfylmx@163.com (M.L.); ksfytgk@163.com (G.T.); ksfylfy@126.com (F.L.); ksfypy@163.com (H.W.); 2 Potato Biology and Genetics Key Laboratory of Ministry of Agriculture and Rural Affairs of the People’s Republic of China, Qiqihar 161000, China; 3 National Potato Germplasm Resources Test-Tube Seedling Library (Keshan), Qiqihar 161000, China

**Keywords:** potato, nitrogen, transcriptome, WGCNA, co-expression, tuberization, nitrogen use efficiency

## Abstract

Background/Objectives: Nitrogen, as an indispensable macroelement for plants, is essential for tuber development. The objective of the present study was to ascertain the key factors underlying nitrogen regulation of potato tuber formation. Methods: The potato variety Kexin 37 was used as the material, and nitrogen deficiency, normal nitrogen level and excessive nitrogen level were employed as treatments, respectively. The response of potato tuber formation to nitrogen was systematically analyzed from the perspective of physiology and transcriptomics. Results: Nitrogen deficiency led to the thickening of the cell wall and plasma membrane, an increase in intercellular space and a decrease in mitochondria in the stolon. The plant height, chlorophyll content, dry matter quality and nitrogen accumulation were significantly reduced, and the number of tubers per plant, tuber weight per plant and commodity rate were significantly reduced. Excessive nitrogen application resulted in late maturity of plants and excessive formation of small potatoes. Transcriptome analysis revealed that differentially expressed genes related to nitrogen stress were mainly enriched in pathways associated with material transport, cell division and carbohydrate metabolism. In addition, there are a series of hub genes in response to nitrogen stress, including polyubiquitin-like, auxin response factor 7-like and protein RRP6-like 2. By constructing a co-expression network, transcription factors (TFs) such as C2H2, WRKY and ARF are involved in regulating tuber formation. Conclusions: The present study constitutes an investigation into the identification of hub genes and potential pathways associated with the formation of potato tubers under varying nitrogen conditions. It provides new insights for further study on enhancing nitrogen use efficiency in potato.

## 1. Introduction

Potato (*Solanum tuberosum* L.) is the third most widely cultivated crop in the world after wheat and rice. Its tubers are rich in nutrients such as starch, protein, vitamins and mineral [1]. The tuber is the storage and harvest organ of potato, which is differentiated from stolon tissue and is the main source of carbohydrates [2]. Its growth and structural differentiation are key biological processes that determine tuber initiation time and yield formation [3]. Therefore, the formation process of potato tubers is one of the decisive events affecting the economic benefits of potato tubers [4]. The regulation of tuber formation is affected by factors such as the environment, hormones, carbohydrates and signal molecules [5,6,7]. It is of great significance for the development of the potato industry to deeply explore the influence of environmental stress on the development of stolons.

Nitrogen (N) participates in the biosynthesis of proteins, nucleic acids, enzymes and other biologically active substances and thus regulates cell division, tissue growth and material metabolism [8]. The supply of nitrogen can affect the yield and production of crops, which is essential for plant life activities [9]. Among the various mineral nutrients absorbed by potatoes, nitrogen accounts for a very high proportion and has an important impact on the formation and development of tubers [10]. Excessive nitrogen application and a reduction in crop nitrogen efficiency will lead to serious environmental pollution problems [11]. Therefore, studying nitrogen response characteristics and tuber formation in potato is of great importance for enhancing nitrogen use efficiency.

The formation of potato tubers mainly encompasses three phases: the occurrence and elongation of stolons, the subsequent bending of the top into the hook, and the expansion of the subapical region to form a tuber [7,12]. It has been reported in earlier studies that the application of a nitrogen concentration of 22.5 mM at the early stage of growth can significantly increase the number of stolons in early-maturing potato, but the application of 37.5 mM nitrogen at the middle and late stages of growth will slow down the rate of tuber formation, reduce the accumulation of photosynthetic capacity, and inhibit the development of the stolon and tuber [13,14]. The accumulation of photosynthetic products is closely related to tuber formation. Reasonable nitrogen fertilizer management is conducive to an increase in potato total starch and amylopectin content [15]. Different nitrogen application rates will regulate potato root yield, starch yield and starch physicochemical properties and affect tuber formation [16]. The nitrogen form is a critical determinant influencing potato tuber quantity [17]. Studies have found that by reducing the ratio of nitrate nitrogen and ammonia nitrogen in the medium, the number and diameter of microtubers formed from test tube seedlings decreased. Compared with nitrate nitrogen treatment, the number and length of stolons treated with ammonia nitrogen increased significantly and the tubers were formed earlier and the number was relatively higher [18]. In addition, key genes related to nitrogen metabolism, such as nitrate reductase (NR), glutamine synthetase (GS), glutamate dehydrogenase (GDH) and nitrite reductase (NIR), are known to directly affect nitrogen uptake and utilization in plants [19]. Previous studies have included a lot of research on the effect of nitrogen on potato tubers. However, the molecular mechanism behind the regulation of potato formation by nitrogen is still unclear. Therefore, it is necessary to further explore the key genes and metabolic pathways in the stolon of potato and understand nitrogen responsiveness and organic phenomena in potato for the selective breeding of potatoes with high nitrogen use efficiency.

However, the physiological and molecular mechanisms underlying nitrogen-regulated potato tuberization from stolon development to tuber formation remain largely unclear. In this study, potato plants were subjected to three nitrogen levels (nitrogen deficiency, conventional nitrogen, and excessive nitrogen), and a combination of physiological, biochemical, and molecular approaches was used to reveal the regulatory roles of nitrogen concentration in tuber formation. Using physiological measurements, RNA sequencing (RNA-seq), and weighted gene co-expression network analysis, we identified key genes, gene modules, and metabolic pathways involved in nitrogen-mediated tuberization. These results provide new insights into the adaptive responses of potato stolon-to-tuber development to different nitrogen regimes and offer a theoretical basis for improving nitrogen use efficiency in potato production.

## 2. Materials and Methods

### 2.1. Plant Materials and Experimental Treatments

The experiment was carried out in the experimental base of Keshan Branch of Heilongjiang Academy of Agricultural Sciences (Qiqihar, China). The tested potato variety was Kexin 37, and its growth period was 66 days. Healthy potato tubers with uniform size and plump buds were sown on May 11, and the plants entered the early stage of tuber formation at 20 days after sowing, with the cultivation environment controlled at 60% relative humidity and a 14 h light/10 h dark photoperiod. The experiment utilized black soil as the test substrate.

The test used a plastic cultivation basin with a volume of about 19.2 L, and 5 drainage holes were set in the bottom of the basin. The same weight of black soil was put into each plastic pot (15 kg), and one seed potato was planted per pot. The main nutrient contents of black soil were as follows: organic matter 86.4 g/kg, total nitrogen 0.206%, total phosphorus 0.032%, total potassium 2.05%, hydrolytic nitrogen 194.4 mg/kg, available phosphorus 12.2 mg/kg, available potassium 299 mg/kg and pH 6.53. Three nitrogen treatments were imposed on potato plants: N0 (nitrogen deficiency), N1 (normal nitrogen, CK), and N2 (sufficient nitrogen). Urea was applied at 0, 6.52, and 13.04 g per pot, equivalent to 0, 373, and 746 kg N/ha, respectively. All plants received a uniform phosphorus and potassium supply: 4.5 g concentrated superphosphate (P2O5: 46%) per pot and 6 g potassium sulfate (K2O: 50%) per pot. After 20 days of growth period, fresh stolon and functional leaf samples from each treatment group were collected and frozen in liquid nitrogen and stored in an ultra-low-temperature refrigerator at −80 °C for subsequent physiological and biochemical parameters and transcriptome determination.

### 2.2. Physiological and Biochemical Parameter Assays

Six healthy and uniform plants were randomly selected from each replicate for the determination of growth and physiological traits. The plant height of potato was determined by the direct measurement method. The determination of leaf chlorophyll content was conducted in accordance with the spectrophotometric method that was previously outlined by Yu et al. [20]. Plant total nitrogen content was assayed via the Kjeldahl method [21], and plant nitrogen accumulation was calculated using the following formula: Plant nitrogen accumulation (g) = Plant total nitrogen content × Plant dry matter weight. Ultrastructures of potato stolon cells were observed according to the method described by Ding et al. [22].

### 2.3. Transcriptomic Analysis

For RNA-seq experiment, we collected fresh stolons of N0, N1 and N2, with three biological replicates per treatment. Transcriptome sequencing was completed by OE Biotech Co., Ltd. (Shanghai, China). Total RNA was extracted from frozen stolon samples using TRIzol reagent (Invitrogen, Carlsbad, CA, USA) following the manufacturer’s protocol. RNA concentration, purity and integrity were determined using a NanoDrop spectrophotometer and Agilent 2100 Bioanalyzer (Agilent Technologies, Santa Clara, CA, USA). Only samples with RIN ≥ 8.0 were used for cDNA library construction with the VAHTS Universal V6 RNA-seq Library Prep Kit. Library sequencing was conducted on the Illumina NovaSeq 6000 platform (Illumina, San Diego, CA, USA) to produce 150 bp paired-end reads.

Fastp was used to filter the original sequence data to eliminate low-quality readings [23], and HISAT was used to map clean readings to the *Solanum tuberosum* L. reference genome [24]. We computed the Fragments Per Kilobase Million (FPKM) value for each gene as documented earlier [25,26]. A |log2-fold change| > 1 and false discovery rate (FDR) < 0.05 were set as the criteria for differential expression. The detailed analytical pipeline was performed in accordance with the previous description [27].

### 2.4. Weighted Gene Co-Expression Network Analysis (WGCNA)

A scale-free co-expression network associated with phenotypic traits was constructed using WGCNA. A gene expression matrix was generated from the transcript profiles of potato stolons under various nitrogen treatments. The optimal soft threshold was identified with the pickSoftThreshold function, and the blockwiseModules function with a power of β = 28 was applied to calculate the correlation coefficients of the expression matrix. We conducted correlation analysis between plant phenotypic data and the identified modules, with candidate modules selected based on a correlation coefficient threshold of |r| > 0.80. Two modules most related to traits were selected. In order to accurately locate the central gene and explore the interaction between genes, remove the disconnected nodes and visualize the network using Cytoscape (version 3.10.4). Hub genes were screened using the criteria of gene significance (GS) > 0.9 and module membership (MM) > 0.9 [28].

### 2.5. Quantitative Real-Time PCR (qRT-PCR) Analysis

Using Glyceraldehyde-3-phosphate dehydrogenase (GAPDH) as an internal reference gene, nine DEGs were randomly selected for qRT-PCR verification. Specific primers were designed using the NCBI Primer-BLAST online tool, and the relative gene expression level was calculated using the 2^−ΔΔCt^ method [29]. The correlation analysis of qRT-PCR and RNA-seq data (R^2^ = 0.9155, *p* < 0.05) (Figure S1) confirmed the reliability of transcriptome sequencing results.

### 2.6. Measurement of Yield and Yield Components

A completely randomized design was applied to the nitrogen treatments (nitrogen deficiency, conventional nitrogen, and excessive nitrogen), with three biological replicates (each replicate containing 15 plastic pots, 1 seedling per pot). For each treatment, 10 mature potato plants (one plant per pot) were randomly selected across all replicates to determine the number of tubers per plant and fresh weight of tubers per plant.

### 2.7. Statistical Analysis

Data statistical analysis was carried out using IBM SPSS Statistics 26.0. Before one-way ANOVA, the Shapiro–Wilk test and Levene’s test were used to evaluate data normality and homogeneity of variance, respectively. Differences among the three treatments were compared by Duncan’s multiple range test at the *p* < 0.05 level, and all data were expressed as mean ± standard deviation (SD).

## 3. Results

### 3.1. Morphological Responses of Potato Plants to Various Nitrogen Supply Levels

**Figure 1:**
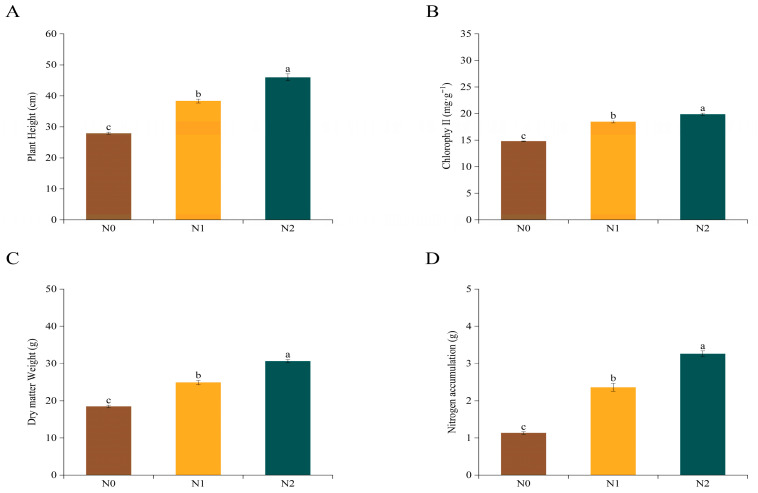
Morphological responses of potato plants to various nitrogen supply levels. (**A**) Plant height, (**B**) leaf chlorophyll content, (**C**) dry matter weight per plant, and (**D**) nitrogen accumulation. All the data are expressed as the means ± SEs, n = 3. According to Duncan’s test, the different lowercase letters indicate significant differences between treatments (*p* < 0.05).

Plant height, leaf chlorophyll content, dry weight, and nitrogen accumulation were measured in potato cultivar Kexin37 under N0, N1, and N2 treatments (Figure 1). Compared with the N1 group, the N0 treatment significantly decreased plant height, chlorophyll content, dry matter weight, and nitrogen accumulation by 27.13%, 19.84%, 25.92%, and 52.05%, respectively. In contrast, the N2 treatment significantly increased these four indices by 19.91%, 7.64%, 23.16%, and 38.30%, respectively, relative to the N1 group.

### 3.2. Ultrastructural Changes in Potato Stolon Cells Under Various Nitrogen Supply Levels

**Figure 2:**
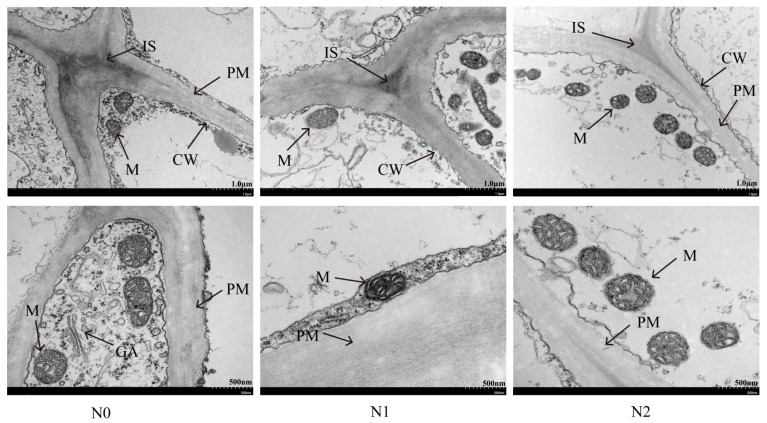
Ultrastructural changes of potato stolon cells under various nitrogen supply levels. CW: cell wall, PM: plasma membrane, M: mitochondrion, IS: intercellular space, GA, golgi apparatus.

Transmission electron microscopy observations revealed that mitochondrial development in potato stolons remained relatively intact under different nitrogen treatments (Figure 2). Mitochondria were distributed adjacent to the cell wall, with a complete structure, spherical morphology, and distinct internal cristae. However, compared with the excessive nitrogen treatment, the non-nitrogen treatment led to a decreased number but an increased volume of mitochondria in potato stolons. Overall, relative to the excessive nitrogen treatment, the non-nitrogen treatment exhibited enlarged intercellular spaces, as well as thickened cell walls and plasma membranes.

### 3.3. Quality Assessments of Transcriptome Sequencing Data

A total of 463.26 M high-quality clean reads were generated, with the Q30 ratio exceeding 93.63% and GC content above 43.49% for all samples (Table S1). These clean reads were mapped to the potato reference genome, with total mapping rates ranging from 82.87% to 89.20%, multiple mapping rates below 8.66%, and unique mapping rates above 76.33%. In total, 23,821 genes were identified. Pearson correlation analysis confirmed high biological repeatability among samples (Figure S2A), and the samples showed good clustering and dispersion (Figure S2B).

### 3.4. Differential Gene Expression Analyses

**Figure 3:**
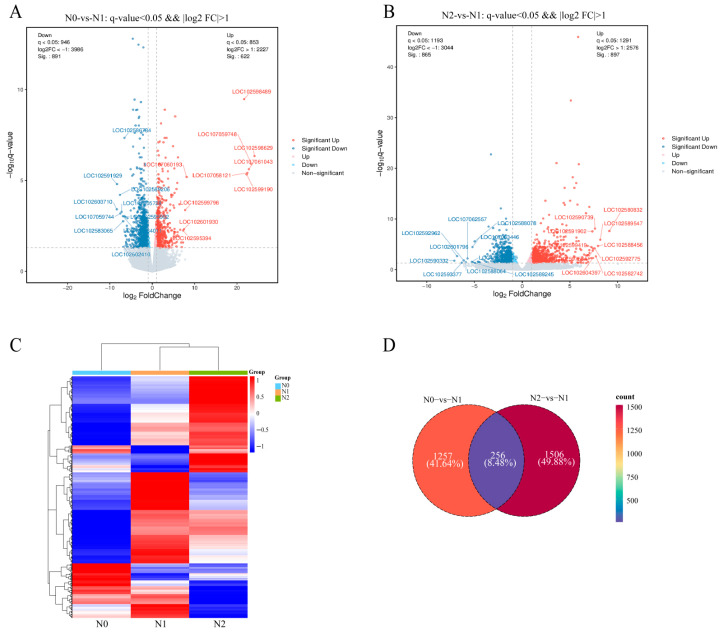
Research overview of stolons transcriptome under different nitrogen levels. (**A**,**B**) Differential expression volcano map. (**C**) Heatmaps of DEGs. (**D**) Venn diagram of DEGs.

A total of 3019 DEGs were identified in the two comparison groups (Table S2). In the N0 vs. N1 comparison, 1513 DEGs were detected, including 622 up-regulated and 891 down-regulated genes (Figure 3A). In the N2 vs. N1 comparison, 1762 DEGs were found, consisting of 897 up-regulated and 865 down-regulated genes (Figure 3B). Cluster analysis was conducted on all DEGs, with the results presented in Figure 3C. Furthermore, a Venn diagram revealed that 256 DEGs were shared between the two groups (Figure 3D), indicating that stolons may regulate the expression of these common genes in response to varying nitrogen conditions.

We performed KEGG pathway enrichment analysis on the 256 common DEGs (Table S9). The top 20 significantly enriched pathways were classified into five functional categories: Cellular Processes (Cell), Environmental Information Processing (Envp), Genetic Information Processing (Genip), Metabolism (Metab), and Organismal Systems (Ouas). Among these, Envp pathways (including MAPK signaling and plant hormone signal transduction) exhibited high enrichment scores; Genip was primarily enriched in protein processing in the endoplasmic reticulum (with the highest enrichment score and number of mapped genes); Metab pathways encompassed terpenoid, carbohydrate, and amino acid metabolism (with sesquiterpenoid/triterpenoid biosynthesis showing high statistical significance); Cell pathways (peroxisome, endocytosis) showed weak enrichment; and Ouas (plant–pathogen interaction) displayed high enrichment scores and significance. Taken together, these shared DEGs in potato stolons under nitrogen treatment were predominantly enriched in hormone signal transduction, protein processing, terpenoid/carbohydrate metabolism, and plant–pathogen interaction pathways, highlighting the critical regulatory roles of these genes in signal transduction, material metabolism, and defense responses to varying nitrogen levels.

### 3.5. GO and KEGG Pathway Analysis of DEGs

**Figure 4:**
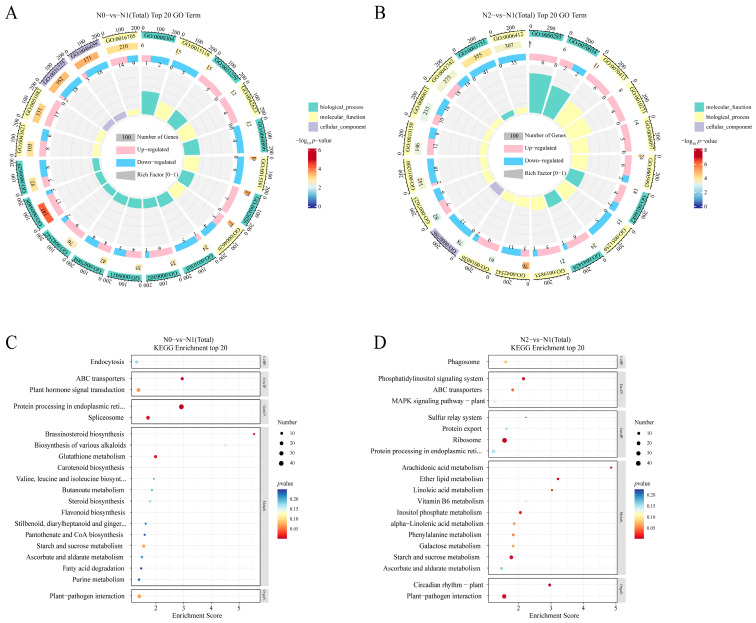
GO and KEGG pathway analysis of DEGs. (**A**,**B**) GO enrichment circular plots (top 20 terms by q-value/*p*-value). (**C**,**D**) Top 20 KEGG enrichment bubble plots.

GO enrichment analysis was performed to reveal the effects of various nitrogen treatments on key biological functions (Table S3). The protein refolding (GO:0042026), anion antiporter activity (GO:0015301) and anchored component of the plasma membrane (GO:0046658) with enriched GO terms were found mainly in N0 vs. N1 (Figure 4A). These functional enrichments reflect adaptive responses to nitrogen deficiency: protein refolding mediates cellular stress repair to mitigate damage from insufficient nitrogen supply, anion antiporter activity facilitates nitrate (the primary nitrogen form absorbed by plants) transport across membranes, and the anchored plasma membrane component maintains membrane structural stability during nitrogen-limited stress. The DEGs were notably enriched in DNA-dependent DNA replication (GO:0006261), the cytokinin metabolic process (GO:0009690), and SNARE complex (GO:0031201) with N2 vs. N1 comparison (Figure 4B), suggesting that excess nitrogen promotes stolon cell proliferation (via enhanced DNA replication) and modulates cytokinin metabolism (a key regulator of stolon development and tuber initiation).

The biological functions of the DEGs were further analyzed via KEGG pathway enrichment. In the N0 vs. N1 comparison, several pathways, including protein processing in the endoplasmic reticulum, ABC transporters, brassinosteroid biosynthesis, spliceosome and glutathione metabolism, were significantly enriched (*p* < 0.05) (Figure 4C). In the N2 vs. N1 comparison, the circadian rhythm–plant interaction, ribosome, starch and sucrose metabolism, plant–pathogen interaction, phosphatidylinositol signaling system, inositol phosphate metabolism and ABC transporter pathways were significantly enriched (*p* < 0.05) (Figure 4D). ABC transporters promote the absorption and transport of nitrogen-containing compounds and secondary metabolites; brassinosteroid biosynthesis regulates stolon growth to adapt to low nitrogen environments, while glutathione metabolism removes reactive oxygen species (ROS) produced during nitrogen deficiency and reduces oxidative damage. Excessive nitrogen can affect tuber formation by regulating carbon metabolism and other pathways.

### 3.6. Co-Expression Network Construction and Module Analysis of Potato Stolons

**Figure 5:**
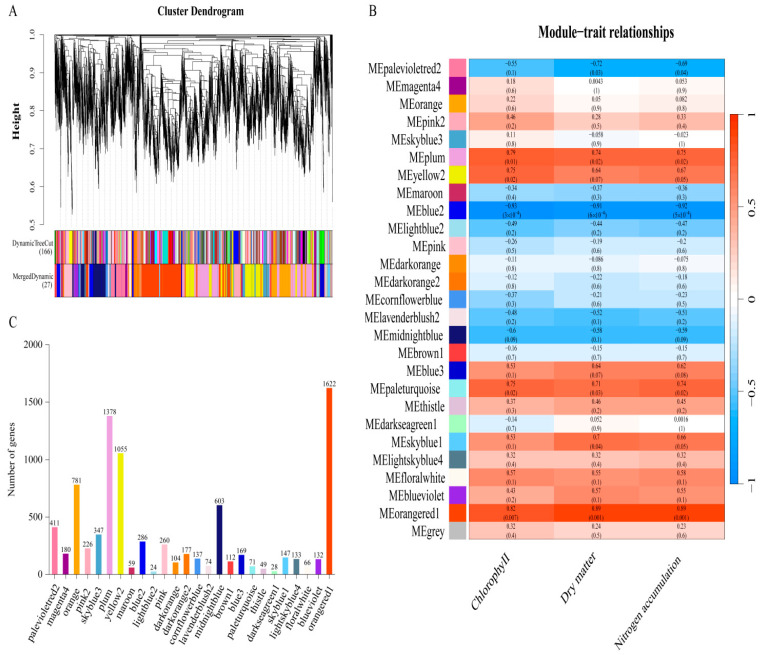
Gene expression clustering and co-expression analysis of transcriptome. (**A**) Clustering tree of co-expression module. (**B**) Heat maps of the correlation between modules and sample traits. (**C**) The number of genes in different modules.

After filtering out genes with low FPKM values, 8667 genes were retained for subsequent analysis. Genes were clustered and assigned into modules using the dynamic tree-cutting algorithm. The module eigengenes were calculated for each module, and similar modules were merged. The branches of the clustering tree represent distinct gene co-expression modules, which are distinguished by different colors. A total of 27 modules were identified, with the gray module representing genes that could not be assigned to any other module (Figure 5A). The number of genes per module ranged from 24 to 1622. The orangered1 module contained the largest number of genes (1622), while the lightblue2 module contained the fewest (Figure 5C).

Subsequently, chlorophyll content, dry matter weight, and nitrogen accumulation (Figure 1) were employed as phenotypic indicators and subjected to correlation analysis with the 27 identified modules. As illustrated in Figure 5B, these three phenotypic traits displayed strong positive associations with the orangered1 module and marked negative correlations with the blue2 module. Specifically, the correlation coefficients of chlorophyll content, dry matter weight, and nitrogen accumulation with the blue2 module were −0.93 (*p* = 3 × 10^4^), −0.91 (*p* = 6 × 10^4^), and −0.92 (*p* = 5 × 10^4^), respectively, whereas those with the orangered1 module were 0.82 (*p* = 0.007), 0.89 (*p* = 0.001), and 0.89 (*p* = 0.001), respectively. Accordingly, these two modules were regarded as key candidate modules for further investigation. A total of 286 genes in the blue2 module and 1622 genes in the orangered1 module were retained for subsequent functional analysis.

### 3.7. Identification of Hub Genes and Transcription Factors (TFs) in the Candidate Modules

**Figure 6:**
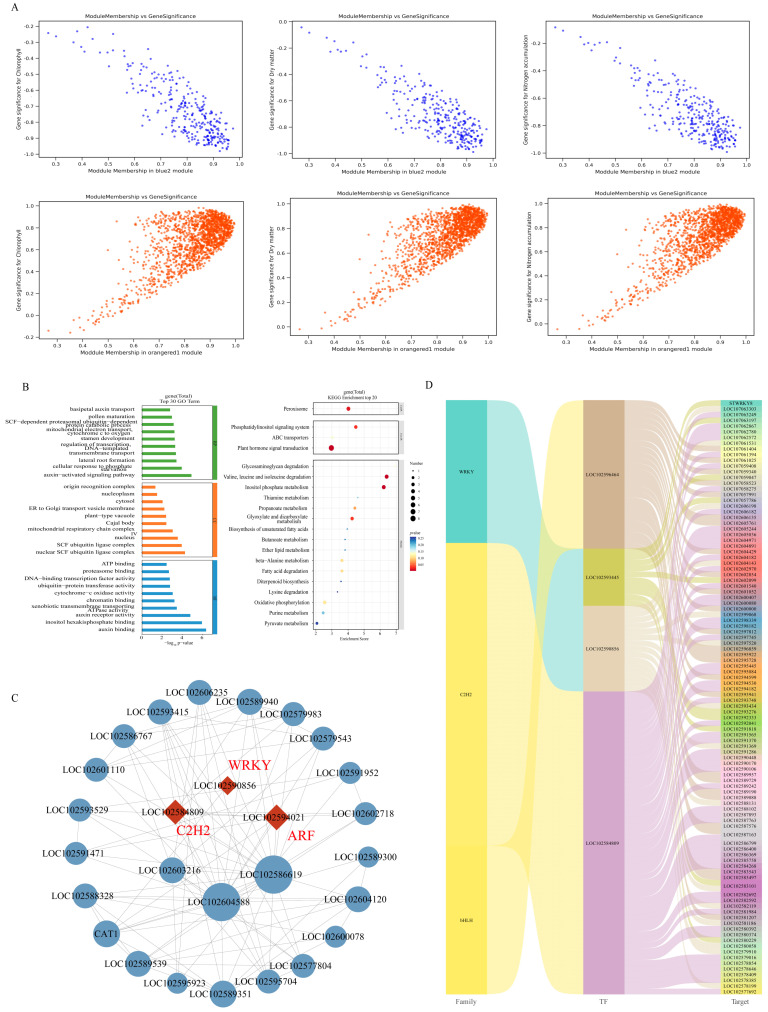
Identification and analysis of key modules of potato stolons under different nitrogen treatments. (**A**) Gene significance (GS) and module membership (MM) relationships between genes and phenotypic traits in the two candidate modules. (**B**) GO and KEGG enrichment analysis of key genes. The ordinate represents the name of the GO term, and the abscissa shows the negative common logarithm of the *p* value derived from enrichment analysis, i.e., the -log10-*p* value, for each annotation. BP: biological process CC: cellular component MF: molecular function. (**C**) The co-expression network and hub gene identification. (**D**) Transcription factor family–transcription factor–target gene Sankey diagram.

The correlations between module membership (MM) and gene significance (GS) for the two key modules are presented in Figure 6A (Table S4). To further refine the candidate gene set, thresholds of MM > 0.9 and GS > 0.9 were applied in the WGCNA analysis. In total, 173 and 8 key hub genes were identified in the blue2 and orangered1 modules, respectively. These genes are closely implicated in nitrogen-mediated regulation during potato tuber development (Table S5).

We further performed GO analysis on the selected genes in the two modules and found that they were mainly enriched in the pauxin-activated signaling pathway (GO:0009734), cellular response to phosphate starvation (GO:0016036), lateral root formation (GO:0043038), transmembrane transport (GO:0055085), nuclear SCF ubiquitin ligase complex (GO:0043224), and auxin binding (GO:0010011) (Figure 6B). KEGG analysis of two specific module genes showed that they were mainly enriched in valine, leucine and plant hormone signal transduction (Figure 6B, Table S6).

Cytoscape software was used to analyze and visualize the co-expression network. The size of the nodes in the network represents the degree of gene connectivity. The first four genes with the highest connectivity are considered to be hub genes and are located at the center of the network (Figure 6C). Transcription factors are important regulatory proteins in biological processes. Among the key genes screened, most of them interacted with three transcription factor families, belonging to WRKY, C2H2 and b HLH, respectively (Figure 6D). In the co-expression network diagram (Figure 6C), we also identified three TFs (WRKY, C2H2, ARF) that are highly connected to hub genes at the center of the network.

**Figure 7:**
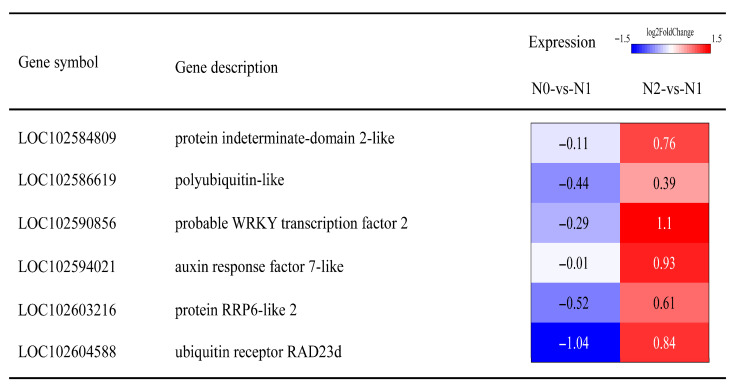
Expression of hub genes.

Given that hub genes and key transcription factors may play pivotal roles in potato adaptation to nitrogen regimes, we focused on these genes for in-depth analysis. As depicted in Figure 7, LOC102586619, annotated as polyubiquitin-like, participates in diverse cellular processes by modulating protein–protein interactions, enzymatic activity, and subcellular localization. LOC102594021, an auxin response factor, regulates the transcription of auxin-responsive genes via direct binding to their promoters and was up-regulated in the N2 vs. N1. LOC102603216, annotated as protein RRP6-like 2, showed up-regulation in N2 vs. N1. but down-regulation in N0 vs. N1. LOC102604588, annotated as ubiquitin receptor RAD23d, is involved in the cellular ubiquitination pathway and displayed elevated expression under high-nitrogen conditions but reduced expression under nitrogen deficiency. Furthermore, two key transcription factors, WRKY (LOC102590856) and C2H2 (LOC102584809), were identified, both of which play essential regulatory roles in plant stress tolerance, growth, and development.

### 3.8. Changes in Potato Yield and Yield Components Under Different Nitrogen Levels

As shown in Table 1, the N2 treatment significantly increased the number of tubers per plant by 36.01% compared with the N1 treatment. In contrast, tuber weight per plant was significantly reduced by 48.06% under N0 and 41.00% under N2, respectively, relative to the N1 treatment. The commercial tuber rate in the N0 and N2 groups was markedly decreased by 33.15% and 36.15%, respectively, compared with N1. In addition, tuber dry weight per plant was significantly lower in the N0 and AN groups, with reductions of 37.71% and 30.91%, respectively, compared to the N1 group.

## 4. Discussion

Nitrogen represents an essential macronutrient for plant growth and development and also plays a critical role in crop protein synthesis and yield formation [30,31]. The occurrence of stolon is the basis of tuber formation [32]. Tuberization was ultimately induced by the increases in number and volume of pith, circular pith, and cortical cells in the proximal region of the stolon base [3]. Our results revealed that nitrogen deficiency altered the ultrastructure of stolon cells in potato. The thickening of cell walls will limit cell expansion and assimilation transport, hindering tuber expansion. The thickening of the plasma membrane will prevent nutrient absorption and signal transduction, interfering with tuber regulatory network. An increase in intercellular space can destroy intercellular connection and transport channels, hindering the accumulation of assimilation, and a decrease in mitochondria will weaken the energy supply. This will eventually lead to tuber initiation failure, swelling blockages, and insufficient dry matter accumulation. However, compared with excessive nitrogen application, nitrogen deficiency led to earlier tuber formation. The reason may be that nitrogen deficiency changed the growth center of potato and then affected the growth of stolon and tuber formation. However, due to the lack of sink organs for accepting and transforming assimilates, the nutrients required for tuber expansion in the later stage are insufficient, which ultimately leads to a significant decrease in the tuber number per plant, tuber weight per plant and commodity rate. The significant increase in plant height and dry matter mass after excessive nitrogen application further confirmed that excessive nitrogen application caused the potato growth center to not be transferred from the aboveground part to the underground part in time, thereby delaying the subapical expansion of stolons and delaying the formation of tubers. This is similar to previous research results [19]. The high nitrogen treatment could not transfer the growth center to the stolon in time, resulting in the late maturity of the plant and the formation of too many small potatoes. However, the chlorophyll content of excessive nitrogen treatment increased significantly, which affected the production and accumulation rate of photosynthetic products, and the sub-apex of stolons also expanded successively. Finally, the number of tubers per plant and the quality of tubers per plant were significantly higher than those of nitrogen deficiency treatment.

As sequencing technologies have advanced, RNA-seq has become a widely used tool for plant functional analysis [33]. We analyzed the potato stolons of three nitrogen treatment groups by transcriptomics, subsequently identifying 3019 DEGs. In this study, GO [34] and KEGG [35] were used to analyze the differential genes, which were mainly enriched in ABC transporters, glutathione metabolism, starch and sucrose metabolism, and the phosphatidylinositol signaling system. These pathways play an important role in material transport [36], cell division [37] and carbohydrate metabolism [38,39] and then regulate the metabolic process of cells, indicating an association between genes and physio-biochemical traits.

For the purpose of identifying gene–trait correlations and exploring the regulatory mechanisms in depth, a systematic WGCNA was performed (Figure 5). The blue2 and orangered1 modules were identified as the key modules. Genes that were highly interconnected within these key modules were defined as hub genes [40]. Previous studies have confirmed that hub genes serve as the core components of co-expression networks and perform essential functions in specific physiological processes [41]. WGCNA was further applied to narrow down the range of candidate genes. By applying the thresholds of MM and GS, 181 candidate genes were obtained, and their correlation was visualized. Among the candidate genes, three genes (LOC102586619, LOC102604588, LOC102603216) were identified as hub genes affecting nitrogen regulation of potato tuber formation according to the connectivity degree. LOC102586619 is considered a polyubiquitin chain. LOC102604588, as a ubiquitin receptor, plays a vital role in the ubiquitin–proteasome system (UPS). UPS is the main pathway of protein degradation in cells [42]. Ubiquitination is a post-translational modification process. Through the catalysis of a series of specific enzymes, ubiquitin molecules label and specifically modify substrate proteins in cells [43]. As a ubiquitin receptor, RAD23d achieves bidirectional regulation of polyubiquitin-like signals through the N-terminal ubiquitin-like domain and the C-terminal ubiquitin-related domain, the UbL domain of ubiquitin receptor RAD23 can directly bind to the proteasome and switch to the ‘substrate escort’ mode under specific conditions (such as cell stress) to deliver ubiquitinated proteins to the degradation pathway [44]. The synergistic effect between the two is an important regulator of plant growth and stress response [45]. Accumulating evidence indicates that the ubiquitin–proteasome pathway serves a critical function in modulating plant organ size, particularly the shape and development of sink organs [46,47,48]. In this study, LOC102586619 and LOC102604588 were down-regulated under nitrogen deficiency and up-regulated under excessive nitrogen conditions, indicating that nitrogen regulates potato tuber formation by affecting the ubiquitination process. Protein RRP6-like 2 belongs to the exonuclease family, which is mainly involved in ribosomal RNA processing and abnormal RNA degradation in the nucleolus to maintain nucleolus homeostasis. Its dysfunction may affect ribosome production, indirectly regulate cell growth and stress response [49]. This study found that Protein RRP6-like 2 showed the same expression pattern as polyubiquitin-like and ubiquitin receptor RAD23d, suggesting that ubiquitin receptor RAD23d may be indirectly associated with Protein RRP6-like 2 through a common stress signaling pathway, protein RRP6-like 2 may regulate the dynamic balance of polyubiquitin-like chains by deubiquitinating enzyme activity, but the specific mechanism is not yet clear.

**Figure 8:**
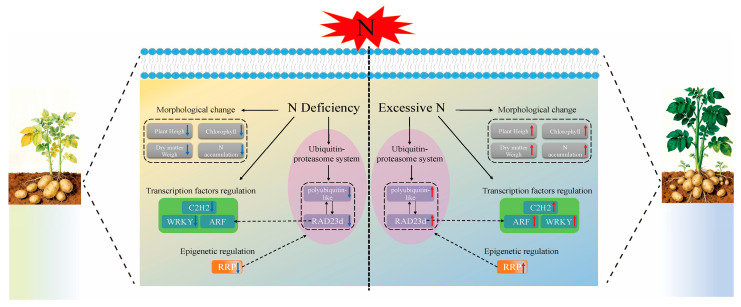
Potential regulatory network of nitrogen regulating potato tuber formation. Red arrows indicate increase or up-regulation; blue arrows indicate decrease or down-regulation. C2H2, protein indeterminate-domain 2-like; WRKY, probable WRKY transcription factor 2; ARF, auxin response factor 7-like; RAD23d, ubiquitin receptor RAD23d; RRP, protein RRP6-like 2.

TFs act as essential regulators in diverse biological processes, including plant growth and development [50,51]. In our study, gene LOC102584809 belongs to the C2H2 family, gene LOC102590856 belongs to the WRKY family, and gene LOC102594021 belongs to the ARF family. C2H2 zinc finger transcription factors are the most widely distributed and well studied zinc finger TFs in plants [52]. C2H2 zinc finger TFs are also involved in the growth and development of many plant organs and structures. More and more studies have shown that C2H2 zinc finger protein plays an important role in plant abiotic stress response and is considered to have the activity of inhibiting abiotic stress [53,54,55]. For example, the overexpression of OsZFP179 enhanced the salt tolerance of rice [56]. Tomato SICZFP1 can induce the expression of related genes in transgenic Arabidopsis and rice under low-temperature stress, thereby enhancing cold tolerance [57]. WRKY transcription factor is named because its WRKY domain contains a highly conserved ‘WRKYGQK’ site, which regulates target gene expression through specific sequences of W-box elements [58]. WRKY proteins play crucial roles in plant responses to abiotic stress. Previous studies have demonstrated that MdWRKY115 enhances drought and osmotic stress tolerance in apples by directly binding to the MdRD22 promoter [59]. TaWRKY31 enhances plant drought resistance by scavenging reactive oxygen species, decreasing stomatal aperture, and modulating the expression of stress-associated genes [60]. TaWRKY17 improves its anti-oxidative stress ability by regulating the expression of abscisic acid (ABA)/ROS-related genes and stress-responsive genes [61]. In this study, we identified co-expressed hub genes from the C2H2 and WRKY families via WGCNA, indicating that these two transcription factor (TF) families may achieve functional crosstalk in regulating potato tuber formation through shared downstream target genes or coordinated signal transduction pathways [62,63]. ARF is the core transcription factor of the plant auxin signal, which plays an important role in the regulation mechanism of plant growth and development [64]. Auxin receptor protein TIR1/AFB, AUX/IAA protein and auxin response factor are involved in auxin signal transduction [65]. ARF has the ability to bind to auxin response elements (AuxREs) and affect plant growth and development by activating or inhibiting the expression of auxin response genes (such as Aux/IAA, GH3 and SAUR) [66]. We found that excessive nitrogen resulted in up-regulation of ARF expression, and the key genes identified by WGCNA were significantly enriched in plant hormone signal transduction pathways. It shows that nitrogen regulates the tuber formation process by affecting plant hormone signals. Previous studies have confirmed that AUX/IAA proteins inhibit ARF activity, and the degradation of AUX/IAA proteins is strictly dependent on the ubiquitin–proteasome system (UPS) [67]. Notably, UPS-mediated substrate degradation often involves competitive binding of ubiquitin receptors or enzymatic components [68,69]. Thus, the UPS-dependent degradation of AUX/IAA may compete with RAD23-mediated ubiquitination for common UPS components (such as proteasomal subunits or ubiquitin-conjugating enzymes), though this competitive interaction requires further biochemical validation. Finally, qRT-PCR was performed to validate the reliability of the transcriptome data. It is speculated that these hub genes and TFs serve as core regulators in the transcriptional network, regulating the expression of potato tuber formation-related genes to respond to nitrogen stress. Further experimental validation is required to confirm the molecular functions of these genes. On this basis, a potential regulatory network governing nitrogen regulation of potato tuber formation was proposed (Figure 8).

Although some insights have been gained from this study, some limitations should be recognized. First of all, the functional roles of identified core genes and TFs need further experimental verification to confirm their specific functions in nitrogen regulation of tuber formation. Secondly, although we hypothesized potential competitive relationships within UPS and functional crosstalk between TF families, these speculations were supported by indirect evidence and published literature, rather than direct biochemical experiments (such as co-immunoprecipitation, in vitro ubiquitination assays) to elucidate molecular interactions.

## 5. Conclusions

In this study, we investigated the response of potato tuberization to nitrogen supply at physiological and transcriptomic levels and identified candidate genes involved in nitrogen-regulated tuber formation. The results showed that nitrogen deficiency could lead to the thickening of cell wall and plasma membrane of stolons, the increase of intercellular space and a decrease in mitochondria, which led to the premature formation of tubers, but the number of tubers per plant, the weight of tubers per plant and the commodity rate were significantly reduced. Excessive nitrogen can significantly increase plant height, chlorophyll content, dry matter quality and nitrogen accumulation but delay the formation time of potato tubers and form too many small potatoes. A total of 3019 differentially expressed genes were screened by transcriptome analysis. WGCNA analysis further identified hub genes involved in nitrogen regulation of potato tuber formation. Polyubiquitin-like, auxin response factor 7-like, and protein RRP6-like 2 may be key functional genes affecting tuber formation. Their expression level may be regulated by TFs such as C2H2, WRKY, and ARF. The hub genes and key TFs identified in this study can be used as molecular markers for breeding programs. By aggregating these favorable genetic loci, we can cultivate varieties with enhanced nitrogen response, balanced vegetative growth and tuber development, stable tuber yield and a high commodity rate under different nitrogen conditions, reduce the dependence on excessive nitrogen fertilizer, and promote the sustainable production of potatoes. However, the regulatory networks underlying plant growth are highly complex, and their precise regulatory mechanisms remain largely elusive and require further investigation.

## Figures and Tables

**Table 1:** Changes in potato yield components.

Treatment	Tuber Number Per Plant	Tuber Weight Per Plant (g)	Commodity Rate > 50 g (%)	Tuber Dry Weight Per Plant (g)
N0	6.67 ± 0.67 b	192.37 ± 4.83 c	53.63 ± 1.91 b	53.80 ± 0.72 b
N1	8.33 ± 0.33 b	370.37 ± 6.66 a	80.22 ± 3.23 a	86.37 ± 2.85 a
N2	11.33 ± 0.88 a	218.50 ± 9.83 b	51.22 ± 0.64 b	59.67 ± 1.75 b

Note: Commercial potato rate = commercially acceptable tuber weight per plant (>50 g tuber weight)/total tuber weight per plant × 100%. All the data are expressed as the means ± SEs, n = 10. According to Duncan’s test, the different lowercase letters indicate significant differences between treatments (*p* < 0.05).

## Data Availability

The RNA-seq data reported in this paper have been deposited in the Genome Sequence Archive at the BIG Data Center (http://bigd.big.ac.cn/gsa, accessed on 29 November 2025), Beijing Institute of Genomics (BIG), Chinese Academy of Sciences, under accession nos. CRA032046. All the data generated or analyzed during this study are included in this published article and its Supplementary Information Files.

## Supplementary Materials

The following supporting information can be downloaded at: https://www.mdpi.com/article/10.3390/genes17030308/s1, Figure S1. Comparison of the RNA-seq result with qRT-PCR. Nine different genes were randomly selected, and the results were converted to log2 FC. Figure S2. Quality Assessments of Transcriptome Sequencing Data. (A) Pearson correlation coefficient analysis. The abscissa and ordinate represent the sample name, and the color represents the correlation coefficient. (B) Boxplot of the FPKM values. The abscissa represents the sample name, and the ordinate represents the commonly used logarithmic transformation value of the FPKM, i.e., log10 (FPKM). The boxplot of each region corresponds to five statistics (maximum, third quartile, median, first quartile and minimum from top to bottom). Table S1. Data information of potato stolon transcriptome. Table S2. The detailed information of DEGs. Table S3. Data information of Gene Ontology (GO) enrichment analysis of DEGs. Table S4. Gene significance (GS) and module membership (MM) relationships between genes and phenotypic traitsin the two candidate modules. Table S5. The threshold values of MM > 0.9 and GS > 0.9 were set for the genes obtained in the blue2 and orangered1 modules. Table S6. GO and KEGG enrichment analysis of key genes. Table S7. qRT-PCR primers for candidate genes. Table S8. Effects of different nitrogen concentrations on plant height, leaf chlorophyll content, dry matter weight per plant, and nitrogen accumulation. Table S9. KEGG enrichment analysis of 256 common differential genes.
