# Supplementary material for: Elucidation of Response Mechanism of Potato to Nitrogen Stress by Physiological and Transcriptional Analyses
Source: Genes (Basel). 2026 Mar 5;17(3):308. doi: 10.3390/genes17030308 (PMC13026097; doi:10.3390/genes17030308)
Supplement: Supplementary file 1 [file genes-17-00308-s001.zip › Supplementary Material Figure.pdf]

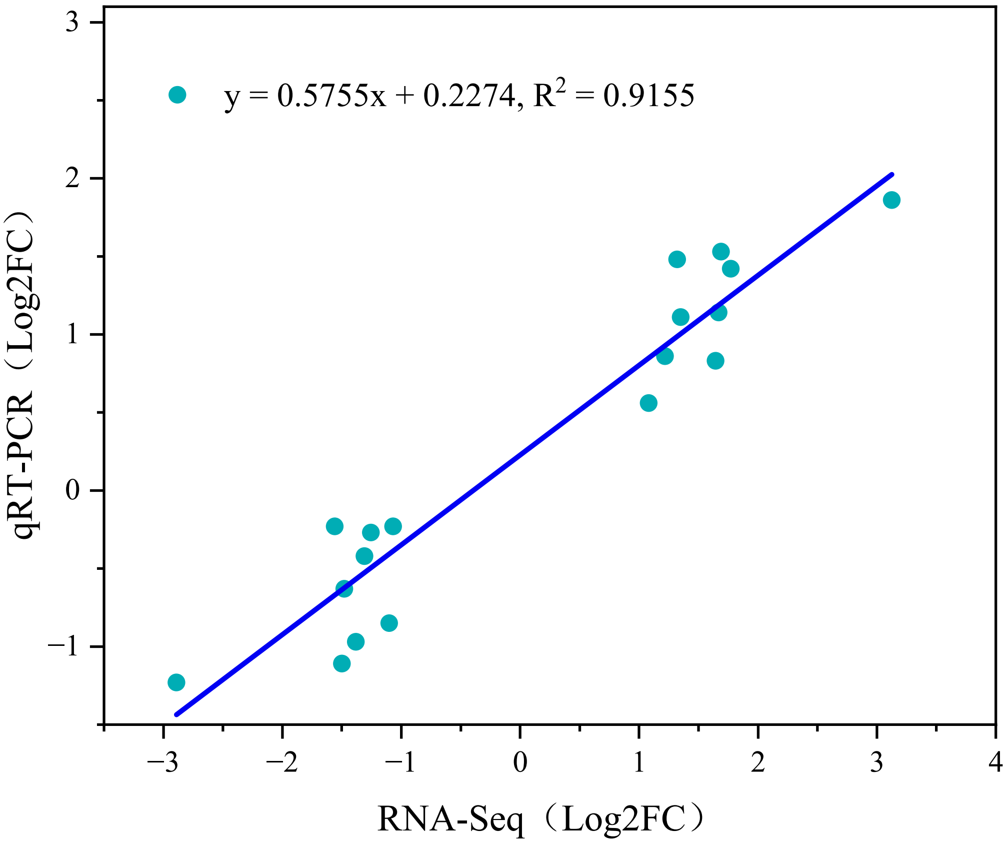


**Fig. S1. Comparison of the RNA-seq result with qRT-PCR.** Nine different genes were randomly selected, and the results were converted to log2 FC.

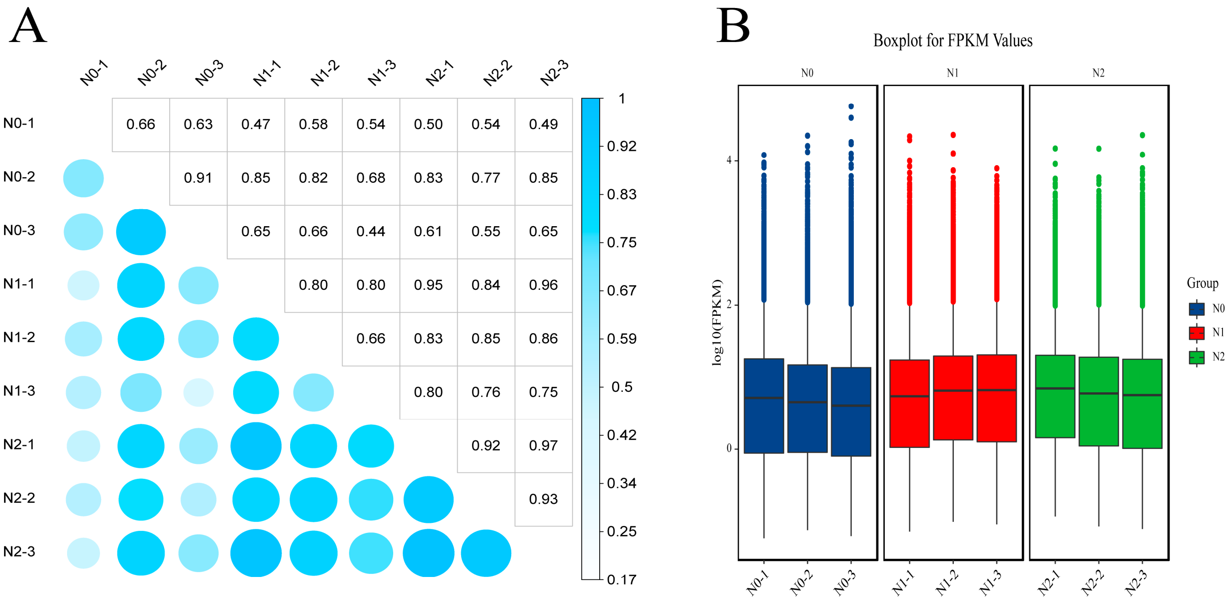


**Fig. S2. Quality Assessments of Transcriptome Sequencing Data.** (A) Pearson correlation coefficient analysis. The abscissa and ordinate represent the sample name, and the color represents the correlation coefficient. (B)

Boxplot of the FPKM values. The abscissa represents the sample name, and the ordinate represents the commonly used logarithmic transformation value of the FPKM, i.e., log10 (FPKM). The boxplot of each region corresponds to five statistics (maximum, third quartile, median, first quartile and minimum from top to bottom).
